# Novel genome editing approaches to manipulate apical meristem activity for crop yield

**DOI:** 10.3389/fpls.2026.1743528

**Published:** 2026-03-25

**Authors:** Elsa Carrasco, Jose Gutierrez-Marcos

**Affiliations:** School of Life Sciences, University of Warwick, Coventry, United Kingdom

**Keywords:** cis-regulatory engineering, CRISPR/Cas applications, domestication, epibreeding, KNOX-cytokinin, meristem determination, shoot apical meristem (SAM)

## Abstract

Meristem function underlies organogenesis and yield potential in crop species, and its regulation depends on the crosstalk of genetic and hormonal networks that balance stem-cell niche maintenance and differentiation. During the shoot apical meristem (SAM) transition, developmental reprogramming shifts the meristem from a vegetative to a reproductive state, referred to as inflorescence meristem (IM). Major regulatory events in this transition include the cytokinin–gibberellin crosstalk, that regulate the expression of the *CLAVATA/WUSCHEL* (*CLV/WUS*) negative feedback loop and key transcription factor families like *KNOTTED-LIKE HOMEOBOX* (*KNOX*) and *SHOOT MERISTEMLESS* (*STM*). Despite the basic principles of apical meristem differentiation are well-described nowadays, major phenotypic bottlenecks were reached in major staple crops during the artificial selection process, known as domestication, leading to a final reduction in total crop yield. This review aims to describe the key processes and genes that play a role in this transition and how they can be artificially targeted to overcome these limitations. Major bioengineering approaches are covered, ranging from classical random mutagenesis with chemicals like ethyl methanesulfonate (EMS) to targeted genome editing using diverse Clustered Regularly Interspaced Short Palindromic Repeats and CRISPR-associated proteins (CRISPR/Cas) systems. Finally, emerging strategies such as epibreeding are considered as promising tools to achieve precise, reversible modulation of meristem activity and to unlock new routes for crop yield enhancement.

## 1 Introduction

Unlike animals, which complete most organogenesis during embryonic development, plants possess a remarkable ability to generate new vegetative and reproductive organs throughout their lifespan (Greb and Lohmann, 2016; Cao et al., 2019; Karami et al., 2023). This continuous regeneration potential gives them the capacity to adapt morphologically to several challenges such as fluctuating environmental conditions or damage caused by herbivores or abiotic stress (Shin et al., 2020). It is sustained by apical meristems, specialised primary growth centres composed of self-renewing pools of pluripotent stem cells at shoot and root tips as well as in axillary positions (Barton, 2010). These structures provide an ongoing source of undifferentiated cells that can be recruited into diverse developmental pathways, thereby enabling the continuous formation of new organs. This developmental plasticity is a defining feature of higher plants and forms the biological foundation for their indeterminate growth and reproductive flexibility. As such, the activity and regulation of apical meristems are central to plant morphogenesis and directly relevant to crop productivity.

The shoot apical meristem (SAM), positioned at the tip of the shoot axis, functions as the primary source of cells for aerial organ initiation. It maintains a central population of undifferentiated stem cells while supplying differentiating progenitors to peripheral regions, where they initiate organ primordia. Upon the floral transition, the SAM is developmentally reprogrammed to form inflorescence meristems (IMs), which subsequently generate floral meristems (FMs) and ultimately reproductive structures such as fruits or grains (Bartlett and Thompson, 2014; Wang et al., 2021). This direct developmental trajectory from SAM to reproductive output further establishes the meristem as a key determinant of yield potential. The root apical meristem (RAM) shares conserved structural and regulatory principles with the SAM but governs root elongation, branching, and nutrient acquisition (Xue et al., 2020; Desvoyes et al., 2021). Different processes play a role in meristem differentiation, including hormone signalling networks, genetic regulatory pathways, and epigenetic inheritance mechanisms (Gaillochet et al., 2017; Nguyen and Gutzat, 2022), most of which have been primarily described in the model dicot organism *Arabidopsis thaliana*. Significant gaps remain in translating this knowledge to economically important eudicot crops. Major species, like maize (*Zea mays*) or rice (*Oryza sativa*) have undergone extensive artificial selection during the domestication process. Despite its role in fixing agriculturally advantageous traits, the domestication programme also led to genetic bottlenecks that constrained allelic diversity, leading to phenotypic modifications such as reduced shoot branching and altered seed-shattering patterns (Doebley et al., 2006).

These traits were historically advantageous for cultivation and harvest efficiency; however, they may now limit yield potential under the pressing demands of modern agriculture. Current projections estimate that global food demand will increase by 35% to 56% by 2050, while the proportion of the global population at risk of hunger is projected to change by between a 91% reduction and an 8% increase relative to current levels, rising to a 30% increase when climate change impacts are taken into account (van Dijk et al., 2021). Within this context, engineering cultivars with an increased number of meristems, particularly inflorescence-producing SAMs and axillary meristems (AMs), has emerged as a promising strategy to enhance reproductive output and contribute to global food security.

While conventional breeding strategies such as hybridisation, marker-assisted selection, and recurrent selection, have undoubtedly improved crop yield over recent decades, their capacity to meet future production demands is inherently limited (Fedoroff, 2010). These approaches primarily rely on naturally occurring genetic variation and incremental selection cycles, which restrict their ability to introduce or precisely modulate complex developmental traits such as meristem activity. Nevertheless, the accelerating demands of global food production, coupled with the challenges posed by climate change and finite arable land, underscore the need for more precise and efficient approaches to enhance crop productivity (Myers et al., 2017; van Dijk et al., 2021).

Another layer of complexity in the context of meristem development is the genetic and functional redundancy among key regulatory genes, as overlapping roles often diminish the impact of single-gene modifications. Although advances in genome editing and synthetic biology have shown promise in modulating meristem traits, the efficiency, reproducibility, and long-term stability of these modifications across diverse crop species remain uncertain. Furthermore, the scarcity and novelty of technologies like high-resolution, single-cell analyses of meristem differentiation in crops limits our mechanistic understanding of how individual cell populations contribute to organogenesis (Sun et al., 2024). Addressing these gaps is critical for optimizing meristem-targeted biotechnologies for agricultural applications.

This review aims to provide a comprehensive overview of apical and shoot meristem manipulation in plant biotechnology by examining how domestication has influenced meristem function, summarising the molecular mechanisms underlying its maintenance and differentiation, and highlighting useful genome editing techniques in the current agricultural bioengineering paradigm. Additionally, it will discuss the challenges and limitations associated with meristem-based interventions in crops and propose future directions for integrating meristem research into sustainable agriculture and global food security. By bridging fundamental plant biology with practical applications, we will explore innovative strategies to enhance crop productivity through targeted manipulation.

## 2 Shoot apical meristem differentiation and regulation

**Figure 1:**
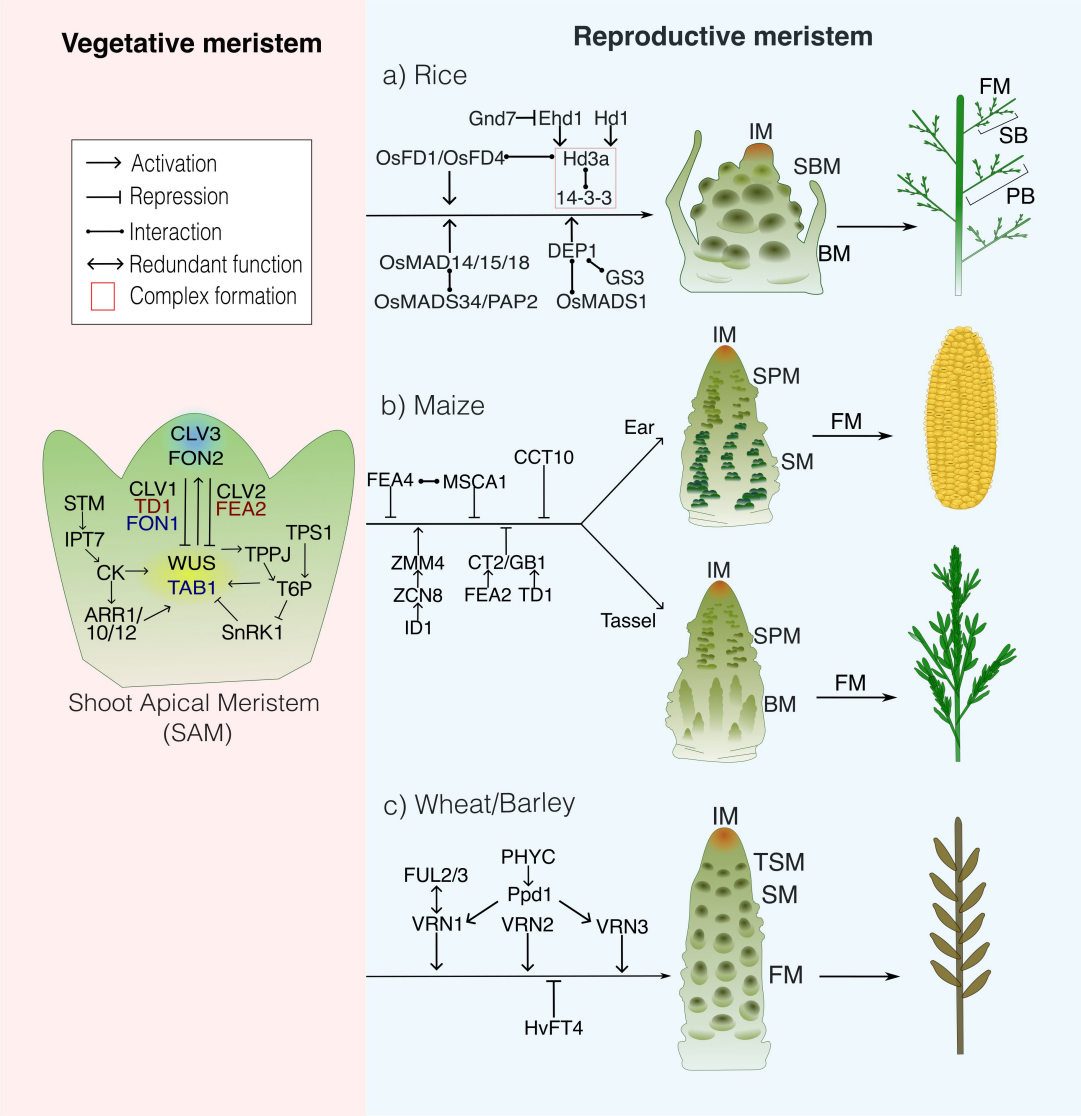
Comparative schematic of major genetic networks regulating the transition from the vegetative shoot apical meristem (SAM) to the reproductive inflorescence meristem (IM) in **(a)** rice, **(b)** maize, and **(c)** wheat/barley. The SAM (left) maintains stem-cell homeostasis through the CLAVATA–WUSCHEL feedback loop, modulated by cytokinin and carbohydrate signalling. Key regulators are shown in black for *Arabidopsis thaliana*, red for *Zea mays* and blue for *Oryza sativa*. During the vegetative-to-reproductive transition, phase-specific regulators (right) coordinate IM establishment and floral determinacy, leading to the final inflorescence structure. SBM, Secondary Branch Meristem; BM, Branch Meristem; SPM, Spikelet Pair Meristem; SM, Spikelet Meristem; TSM, Terminal Spikelet Meristem; FM, Floral Meristem.

The Shoot Apical Meristem (SAM) comprises three histological layers (L1-L3) and overlapping functional domains that work together to sustain the plant’s indeterminate growth. The central zone (CZ) serves as a reservoir of slowly dividing stem cells maintained by signals from the subjacent organising centre (OC); key markers of these areas include *CLAVATA 3* (*CLV3*) in the CZ and *WUSCHEL* (*WUS*) in the OC. Cells displaced laterally enter the peripheral zone (PZ), where polar auxin transport and boundary factors specify organ primordia, while the rib zone (RZ) extending beneath the CZ, drives axial elongation and internal stem tissue production. This spatial logic is highly conserved across angiosperms and is also responsible for key morphological crop traits. Orthologous models in cereals, like *CLAVATA/FLORAL ORGAN NUMBER* (*CLV/FON*) receptors and *KNOTTED1/ORYZA SATIVA HOMEOBOX1* (*KN1/OSH1*) modulate meristem size, inflorescence architecture, and therefore grain number (Wang et al., 2018; Xue et al., 2020) (**Figure 1**).

Additionally, during the vegetative to reproductive transition, leaf-derived florigen: *FLOWERING LOCUS T/TWIN SISTER OF FT* (*FT/TSF*) in *Arabidopsis thaliana* and *HEADING DATE 3a/RICE FLOWERING LOCUS T 1* (*Hd3a/RFT1*) in grasses, a mobile signal encoded by the *FT* gene, is transported to the SAM and AMs, where it triggers transcriptional reprogramming (Tsuji, 2017). This leads to the formation of inflorescence meristems (IMs) and eventually floral meristems (FMs), which will determine the plant’s reproductive output (Wang and Jiao, 2023). Thus, understanding the pathways responsible for these differentiations is key for developing strategies to enhance crop yield.

### 2.1 Hormonal and genetic regulation of SAM activity

SAM activity is sustained by regulatory networks that integrate positional cues, hormone gradients, and gene expression programmes to balance stem cell maintenance with organ initiation. A central feature is the delicate hormone balance between auxin and cytokinin (CK). The latter, produced in the CZ, promotes stem cell proliferation and inhibits differentiation, whilst auxin accumulates in the PZ to induce organogenesis. Within this framework, conserved genetic pathways also coordinate growth across domains, including the *CLV/WUS* feedback loop, *KNOTTED-LIKE HOMEOBOX (KNOX)* transcription factors, and post-transcriptional modifications mediated by small RNAs and RNA binding proteins (RBPs). Together, these regulators provide multiple points of control over meristem function, several of which represent promising targets for crop bioengineering (**Figure 1**).

#### 2.1.1 CLAVATA/WUSCHEL feedback loop and CLE-LRR receptor signalling

At the centre of the *CLV/WUS* negative feedback loop is the transcription factor (TF) *WUS*, part of the *WUS-RELATED HOMEOBOX* (*WOX*) family. This protein maintains the undifferentiated state of overlying stem cells in the OC and moves symplastically via plasmodesmata into the immediate apical layers (Agarwal et al., 2022). When it reaches L1, it directly induces expression of CLV3, a small secreted peptide from the CLV3/EMBRYO-SURROUNDING REGION (CLE) peptide family. CLV3 functions as a signalling ligand perceived by several plasma membrane localised leucine-rich repeat (LRR) receptors. The best-characteried include LRR-kinase CLAVATA1 (CLV1), and CLAVATA2 (CLV2), a LRR-protein that forms a functional complex with the pseudokinase CORYNE (CRN) and RECEPTOR-LIKE PROTEIN KINASE2 (RPK2), also known as TOADSTOOL 2 (TOAD2) (Kinoshita et al., 2010; Fletcher, 2020). Upon perception of CLV3, these complexes activate intracellular signalling cascades, including a mitogen-activated protein kinase (MAPK) pathway, which ultimately represses *WUS* transcription (Betsuyaku et al., 2011). Additionally, signals from the epidermal L1 help anchor the OC at the apex, preserving geometry as the shoot tip grows. The phytohormone CK promotes *WUS* and influences the apical–basal positioning of the OC. Additionally, mobile small RNAs, highlighting *miR394* and *miR171*, restrict stem competence ensuring that only cells at the tip of the CZ respond to *WUS* and maintain undifferentiated cell identity (Knauer et al., 2013; Han et al., 2020).

Plenty of genetic evidence exists to support this model. Loss-of-function mutations in CLV3 or its receptors cause enlarged SAMs due to unregulated *WUS* activity that results in excessive stem cell proliferation and meristem fasciation (Yamaguchi et al., 2017). In a complementary manner, excessive CLV signalling leads to a premature meristem termination through an insufficient maintenance of the stem cell pool. Adding another layer of complexity, *WUS* not only activates CLV3 but also directly represses CLV1 transcription by binding to its promoter (Busch et al., 2010). This dual negative feedback is responsible for avoiding fluctuations in stem cell number and receptor abundance, ensuring robustness in meristem regulation.

Although best studied *in Arabidopsis*, the central regulatory framework of the CLV–WUS pathway is broadly conserved across angiosperms, including cereals. In grasses, however, diversification of meristem types has been accompanied by extensive sub functionalisation and shifts in spatial deployment of key components. In the vegetative SAM of rice and maize, *WUS* orthologues are not maintained in the canonical OC location, whereas in floral meristems their expression more closely resembles the *Arabidopsis* pattern (Knauer et al., 2019; Schlegel et al., 2021). In vegetative apices, *WUS* transcripts are instead associated with leaf-initiation sites or adjacent tissues. This redistribution suggests that *WUS*-related signalling contributes to developmental programmes beyond classical stem-cell maintenance in grasses and that additional factors sustain vegetative meristem homeostasis (Nardmann and Werr, 2006; Satterlee et al., 2020). A plausible interpretation is that sub functionalisation within the WOX family, together with altered CLE/TDIF peptide–receptor deployment in grasses, is buffered by compensatory regulators that stabilise the vegetative SAM.

Genetic analyses in cereals support this view of both conservation and divergence. In maize, *THICK TASSEL DWARF1* (*TD1*) and *FASCIATED EAR2* (*FEA2*) are orthologues of *CLV1* and *CLV2*, respectively, and their disruption causes meristem over proliferation and fasciation. Notably, *FEA2* maps to a quantitative trait locus (QTL) for kernel row number, indicating that natural variation in this pathway has contributed to yield improvement under selection (Taguchi-Shiobara et al., 2001). In rice, *TILLERS ABSENT1* (*TAB1*) interact with *FLORAL ORGAN NUMBER1* (*FON1*) and *2* (*FON2*) to regulate floral meristem size, orthologs for *WUS, CLV1* and *CLV3*, respectively (Tanaka and Hirano, 2020). Collectively, the *CLV–WUS* feedback loop exemplifies a robust and conserved regulatory architecture that integrates positional information, hormonal cues, and metabolic signals to maintain meristem function. Its conservation across diverse plant species, including model organisms, and direct influence on traits linked to yield make it an attractive target for precision breeding and genome editing strategies in crop improvement.

Another layer of complexity in the regulation of this pathway is introduced by trehalose-6-phosphate (T6P) signalling. This key sugar intermediate, synthesised by *TREHALOSE PHOSPHATE SYNTHASE1* (*TPS1*), acts as an integrator of carbon status and developmental signalling and has been directly linked to maintenance of the *WUSCHEL*-mediated stem cell identity at the SAM. In *Arabidopsis*, elevated T6P levels inhibit the energy-sensing kinase SNF1-RELATED KINASE 1 (SnRK1), which under normal conditions represses anabolic metabolism and limits *WUS* expression. Moreover, this TF itself induces expression of *TREHALOSE PHOSPHATE PHOSPHATASE J* (*TPPJ*), which locally reduces T6P concentrations, establishing a double negative feedback loop that fine tunes stem cell homeostasis within the SAM (Fichtner and Lunn, 2021; Musialak-Lange et al., 2025).

#### 2.1.2 KNOX/STM–cytokinin module and auxin–cytokinin crosstalk

Another important family of TFs that interacts with the *WUS–CLV* loop to maintain stem cell homeostasis is the *KNOTTED-LIKE HOMEOBOX* (*KNOX*) group, which is also central to SAM establishment and maintenance (Coudert et al., 2019). In *Arabidopsis*, *KNOX I* factors such as *SHOOT MERISTEMLESS* (*STM*) sustain pluripotency both by interacting with *WUS* at the protein level (Su et al., 2020) and by promoting cytokinin accumulation in the shoot apex, primary through the upregulation of *ISOPENTENYLTRANSFERASE* (*IPT*) genes, including *IPT7*. In parallel, components of the *LONELY GUY* (*LOG*) pathway contribute to CK activation within the same domain (Kuroha et al., 2009). This phytohormone is in turn responsible of activating type-B *ARABIDOPSIS RESPONSE REGULATOR* transcription factors (*ARR1*, *ARR10* and *ARR12*), which bind the *WUS* promoter and directly stimulate its transcription. In addition to transcriptional control, CK signalling enhances WUS protein stability *in vivo*, thereby prolonging its repressing activity within the OC. Perception of the *CLV3* signal by CLV receptors spatially confines *WUS* activity, establishing a boundary that prevents its intercellular spreading (Wang et al., 2017; Snipes et al., 2018; Xie et al., 2018).

In *Zea mays*, where this gene family was initially described, *KNOX* genes like *KNOTTED1* (*KN1*) similarly function to sustain meristem activity and contribute to the formation of various plant structures (Chen et al., 2021). In inflorescences, the KN1 cistrome comprises several thousand genomic *loci*; integration of ChIP–seq with transcriptome data showed that 643 bound genes are transcriptionally modulated in one or more tissues (Bolduc et al., 2012). These direct targets are strongly enriched for TFs, including additional homeobox genes, and for components of hormone pathways (gibberellin, cytokinin and auxin), consistent with KN1 acting near the apex of a hierarchical gene-regulatory network that defines meristem identity and competence. Additionally, *KN1* directly upregulates GA2-oxidase1 (GA2ox1) production, reducing bioactive gibberellin (GA) levels, an effect that favours meristem maintenance by antagonising differentiation cues (Bolduc and Hake, 2009).

An additional key regulator of meristem size in maize is *FASCIATED EAR 4* (*FEA4*), a TGA-class basic leucine zipper (bZIP) TF (*PERIANTHIA-like*). The transcripts of this TF are enriched in the peripheral zone and incipient primordia but reduced in the central stem-cell domain. Consistently, loss-of-function *fea4* mutants produce enlarged, fasciated shoot and inflorescence meristems and display aberrant floral branching, phenocopying the overproliferation phenotypes seen in other CLV-pathway mutants such as *fea2* and *fea3* (Taguchi-Shiobara et al., 2001; Je et al., 2016). This indicates that *FEA4* normally functions to limit meristem proliferation and promote determinacy. At the mechanistic level, this TF binds cis-regulatory elements of auxin-responsive genes and modulates their expression to refine auxin maxima and minima across the peripheral zone, thereby licensing organ initiation sites and sharpening the central-peripheral boundary. In this context, FEA4 potentially acts antagonistically with KN1. Whilst the latter promotes the expression of genes that maintain stem cell populations in an undifferentiated state, FEA4 promotes pathways that lead to differentiation and the termination of meristem activity (Pautler et al., 2015).

Comparable systems to restrict meristem size are conserved across other staple crops. In rice, the cytokinin receptor *HISTIDINE KINASE 4* (*OHK4/OsHK4*), identified through the *panicle length 1* (*pal1*) mutant, operates with *RESPONSE REGULATOR 21* (*OsRR21*), a type-B ARR orthologue, and *IDEAL PLANT ARCHITECTURE 1/WEALTHY FARMER’S PANICLE* (*IPA1/WFP*) in a positive feedback circuit that tunes panicle meristem activity and branching, consistent with the described KNOX→cytokinin→ARR→*WUS* axis constrained by CLV-like signalling (Chun et al., 2023). In barley (*Hordeum vulgare*), CLV/CLE components *CLAVATA1* (*HvCLV1*) and *FLORAL CROWN PEPTIDE1* (*HvFCP1*) delimit inflorescence and rachilla meristem activities, directly paralleling the *Arabidopsis*/maize homeostat and providing a cereal counterpoint to KN1-like pro-meristem inputs (Vardanega et al., 2025). In tomato (*Solanum lycopersicum*), natural variation at *LC/WUS* and *CLV3/fas loci* calibrates floral meristem size and therefore locule number. Regulatory changes near *LC/WUS* elevate *WUS* activity while alterations at *CLV3/fas* weaken CLV-mediated restriction; this WUS-centred promotion balanced by CLV feedback intersects with cytokinin/auxin modules (Baranov et al., 2024; Wang and de Maagd, 2025).

### 2.2 Regulation of SAM specification

In terms of further differentiation, the SAM can be classified into vegetative and reproductive states. The vegetative SAM forms axillary meristems (AMs) at the centre of the axil and ultimately gives rise to structure such as leaves, stems and branches. In contrast, the reproductive SAM, also known as inflorescence meristem (IM), progressively differentiates into floral meristems (FMs) arranged along a stem (Yang et al., 2023).

Both final structures are breeding targets since they ultimately influence crop yield (Wang et al., 2021). The transition of the SAM into IM, and the subsequent elaboration of branch and spikelet lineages, are coordinated by meristem-identity MADS-box factors, florigen/photoperiod signalling, G-protein related pathways, determinacy modules, and a set of patterning pathways like *RAMOSA* (*RA*), *SQUAMOSA PROMOTER BINDING PROTEIN-LIKE* (*SPLs*) or *FRIZZY PANICLE* (*FZP*) TFs, with additional modulation by cytokinin and redox control. In *Arabidopsis* we observe an indeterminate pattern where IMs are constantly producing secondary IMs and FMs, whereas in the *Poaceae* family the IM first generates branch meristem (BM) and several spikelet meristems (SMs), with lineage-specific determinacy, indeterminate in rice and maize panicles (lacking a terminal spikelet), but determinate in *Triticeae* (wheat/barley), where formation of a terminal spikelet fixes the final spikelet number (Wang et al., 2018).

The accumulation of FT-like florigen at the apex and assemble the florigen activation complex (FAC). In rice, Hd3a binds to 14-3-3-protein to form a complex that subsequently recruits bZIP TFs like OsFD1 and OsFD4, providing a direct trigger for SAM→IM reprogramming (Taoka et al., 2011; Cerise et al., 2021). Upstream day-length regulators set FAC timing and thereby modulate IM behaviour. Under short days, Heading date 1 (Hd1) and Early heading date 1 (Ehd1) promote Hd3a (Endo-Higashi and Izawa, 2011). Monocot-specific Ehd2 in rice and INDETERMINATE 1 (ID1) in maize feed into this node, promoting flowering by causing Ehd1 and ZCN8 upregulation respectively (Matsubara et al., 2008). Conversely, Grain number, plant height, and heading date 7 (Ghd7) represses Ehd1 in a phytochrome-dependent manner (Xue et al., 2008) thereby delaying flowering but increasing secondary branching and panicle size in rice. Its maize orthologue while *ZmCCT10*, exhibits comparable effects by remodelling tassel and ear morphology (Stephenson et al., 2019).

In *Triticeae*, homologous photoperiod hubs continue to shape IM output. FT-like signals act downstream of Ppd-1 and PHYC, with FT dosage influencing spikelet initiation and number. Wheat *VERNALIZATION 1, 2 and 3* (*VRN1/2/3*) promote IM transition and spikelet formation. Loss-of-function mutants of these genes delay heading, prolong spike development, and often result in an increased number of spikelets. Notably, *VRN1* and its *FRUITFULL*-like (*FUL*) paralogues *FUL2* and *FUL3* function redundantly across these transitions, as demonstrated by *ful2* null mutants, which display increased florets per spikelet and higher grain number per spike in field conditions (Yan et al., 2004; Yan et al., 2006; Li et al., 2019). In contrast, FT paralogue effects can be antagonistic, as seen with barley *HvFT4* overexpression (Pieper et al., 2021). These flowering regulators remain active beyond the transition itself, continuing to impact IMs development and thereby contributing to yield traits. Meristem-identity MADS-box genes then consolidate and elaborate IM fate. In rice, the family *APETALA1/FRUITFULL* (*AP1/FUL*)-like genes *OsMADS14, OsMADS15* and *OsMADS18* act with the *SEPALLATA* (*SEP*) gene *PANICLE PHYTOMER2* (*PAP2*), also known as *OsMADS34*; combined knock-down delays the SAM to IM transition and can replace a single inflorescence with multiple vegetative shoots, demonstrating a direct requirement for the acquisition of IM identity (Kobayashi et al., 2012).

Parallel signalling modules help calibrate the size and competence of the transitioning apex. A heterotrimeric G-protein pathway intersects genetically with CLV receptors to restrict or expand meristem output at the floral transition. In maize, the Gα protein COMPACT PLANT2 (CT2) acts with FEA2 to regulate SAM development, where constitutively active CT2 results in enlarged ear IMs and an increased spikelet density and kernel row number. Complementary, the Gβ subunit 1 (ZmGB1) operates with CT2 in the same complex during inflorescence development (Je et al., 2018; Wu et al., 2020). In rice and wheat, Gγ subunits DENSE AND ERECT PANICLE1 (DEP1) and GRAIN SIZE 3 (GS3) interact with the E-class factor *OsMADS1* influencing panicle/spike traits (Liu et al., 2018).

Beyond genetic and hormonal regulators, metabolic signalling provides an additional layer of control over meristem architecture during the SAM→IM transition. In *Arabidopsis*, increased T6P levels accompany SAM doming and promote meristem expansion through cytokinin-mediated activation of *WUS*, while low T6P levels or loss-of-function mutations in *TREHALOSE PHOSPHATE SYNTHASE 1* (*TPS1*) constrain SAM size and delay flowering (Fichtner and Lunn, 2021). T6P thus acts as a metabolic proxy for sucrose availability, integrating carbon status with meristem activity via inhibition of the energy sensor SNF1-RELATED PROTEIN KINASE 1 (SnRK1) complex. In grasses, members of the TREHALOSE PHOSPHATE PHOSPHATASE (TPP) family locally modulate this balance to pattern inflorescence architecture. For instance, *RAMOSA3* (*RA3*) in maize and TREHALOSE-6-PHOSPHATE PHOSPHATASE 7 (*OsTPP7*) in rice shape determinacy gradients by reducing T6P in specific domains and altered TPP expression perturbs branch initiation and spikelet density. These findings suggest that spatially regulated T6P turnover coordinates metabolic inputs with developmental signalling networks to calibrate the size, activity, and determinacy of the transitioning apex. In this way, T6P signalling provides a physiological link between carbon availability and the structural elaboration of inflorescence architecture, a process with clear agronomic relevance in yield formation (Paul et al., 2020; Griffiths et al., 2025).

Comparable regulatory principles regulate the establishment of determinacy in axillary meristems (AMs). In grasses, AM fate is determined by a finely tuned balance between branching repressors that maintain dormancy and determinacy-promoting factors that specify reproductive identity. The TCP transcription factor *teosinte branched1* (*tb1*; *Tatb1* in wheat, *Zmtb1* in maize and *INT-C* in barley) acts as a master integrator of hormonal and environmental cues to suppress excessive AM outgrowth (Takeda et al., 2003; Dixon et al., 2018; Wang et al., 2022). In rice, *tb1* represses genes involved in axillary bud activation, including *MADS-box 57* (*OsMADS57*) and *FINE CULM 1* (*OsFC1*), and in wheat interacts with FT1-like proteins to couple branching control with photoperiodic and florigenic signalling (Dixon et al., 2018). Conversely, the *GAI-RGA-and-SCR* (*GRAS*) TFs *MONOCULM1* (*MOC1*) and *MONOCULM3* (*MOC3*) promote AM initiation and tiller bud identity by activating *FLORAL ORGAN NUMBER1* (*FON1*), a *CLV1* orthologue that limits meristem proliferation once AMs are established. Thus, a balanced activity between these regulators is the ultimate determinator of number of initiated tillers, with *moc1* or *moc3* mutants showing a drastic reduction in culm number (Shao et al., 2019). Determinacy of lateral meristems is subsequently reinforced through the activity of MADS-box transcription factors, including *FRIZZY PANICLE* (*FZP*) and *OsMADS34/PAP2*, which as well as IM determinacy promote spikelet meristem identity and suppress further branching in developing panicles. Loss of FZP function results in the reiteration of branch meristem fate, producing highly branched inflorescences, whereas its overexpression accelerates meristem termination. In maize, *BRANCHED SILKLESS1* (*BD1*), an *FZP* orthologue, fulfils an equivalent role in restricting axillary meristem proliferation and spikelet meristem identity (Wang et al., 2023). Taken all together, these shared regulatory mechanisms, including transcriptional, hormonal and metabolic cues, show that a conserved regulatory framework coordinates meristem determinacy across apical and lateral contexts.

Tillering, the process plants undergo to produce lateral branches, in staple crops is an important factor that determines ultimate crop yield, given its direct correlation with AMs initiation and differentiation into panicles. Thus, new approaches aim to modulate this phenotype through reversible, dosage-sensitive mechanisms that mirror natural regulatory plasticity. This has previously been achieved through classic mutagenesis. An example would be the rice gene *MONOCULM1* (*OsMOC1*), that encodes a GRAS-like TF and works with *MONOCULM3* (*OsMOC3*) to activate the *CLV1* homologue *FLORAL ORGAN NUMBER1* (*FON1*). This pair of genes is known for its role in tiller bud formation, as mutations in either *MOC3* or *MOC1* result in plants with a reduced number of culms. After the latter was described to have a direct correlation with *Oryza sativa MADS-box Interacting Protein 1* (*OsMIP1*), targeted manipulation of this gene enabled an increase in final tiller number (Sun et al., 2010; Shao et al., 2019).

## 3 Effects of domestication on crop yield production

The described regulatory mechanisms that govern SAM maintenance and IM/FM differentiation are responsible for key phenotypic shifts in the architecture of staple cereal crops. These changes have occurred over evolutionary time and were further shaped by domestication. While natural selection drove adaptive traits in wild plant populations, artificial selection accelerated and directed this process to favour specific phenotypes (Alam and Purugganan, 2024). By selecting for desirable traits, such as increased organ size, altered branching patterns, or synchronised flowering, humans transformed wild progenitors into crops suited to contemporary agricultural demands. This suite of morphological, physiological, and genetic changes that occurred over a relatively short time, related to other evolutionary processes, is collectively referred to as the domestication syndrome (Di Vittori et al., 2017).

Domesticated cereal grasses typically exhibit a more compact and efficient architecture compared to their wild ancestors, often characterised by fewer and shorter tillers, erect growth, and larger, more harvestable inflorescences (Chen et al., 2021). These characteristics facilitate human cultivation and recollection. A classic example is the domestication of rice (*Oryza sativa*) from its progenitor, *O. rufipogon*, which has given rise to two main subspecies, indica and japonica, both of which were domesticated independently in different regions of Asia. One of the main traits selected is reduced seed shattering, which facilitates natural seed dispersal but complicates harvesting (Gao and Innan, 2008). The genetic basis of domesticated loci has been extensively studied in Asian rice, thanks to a high-quality reference assembly (IRGSP-1.0) and deep resequencing resources, which together permitted fine-mapping of domestication sweeps to causal variants and regulatory elements (Goff et al., 2002; Shang et al., 2023). The loss of seed shattering, a canonical domestication trait, was traced to at least three loci with complementary modes of action. First, *SHATTERING4* (*SH4*), which encodes a trihelix TF, experienced a single-nucleotide polymorphism (SNP) in exon 1 G→T, resulting in a Lys→Asn substitution in the DNA-binding domain that ultimately causes an attenuated abscission-layer differentiation and thereby reduced shattering (Hasan et al., 2023) (Ishikawa et al., 2022). Interestingly, this allele is present at moderate frequencies in wild *O. rufipogon* populations. In the *japonica* subspecies, it is tightly correlated with the non-shattering phenotype, indicating that it underwent positive artificial selection. The comparatively high haplotypic diversity surrounding the locus in wild backgrounds and weaker evidence of a classic hard sweep together suggest that selection likely acted on pre-existing variation rather than on a novel mutation (Zhu et al., 2012).

Similarly, qSH1, which encodes a BEL1-type homeobox TF, carries a regulatory SNP in its 5′ upstream region that eliminates expression in the abscission zone, thereby preventing cell separation and seed release (Konishi et al., 2006). Another TF that contributes to the shattering phenotype is *OsSH1/SH1*, a YABBY family gene that regulates abscission-zone (AZ) formation; both coding and cis-regulatory variants at this locus underlie phenotypic diversity amongst the shattering phenotypes found both across cultivars and in wild specimens. Additionally, within the *OsSh1* region lies *qSH3*, a site where a single base change also affects AZ development (Li et al., 2020; Ishikawa et al., 2022). Genetic and population analyses led to the conclusion that to achieve completely non-shattering phenotypes a combination of the *sh4* coding change with qSH3/OsSh1, while *qSH1* plays a smaller, background-dependent role. Comparative studies in sorghum and maize show that their SH1 genes underwent similar selection during domestication, revealing that tweaking YABBY genes controlling the abscission zone was a common evolutionary route to reduce seed shattering across grasses. Taken together, *sh4*, *qSH1*, *qSH3* and *OsSH1/SH1* show how small coding and regulatory changes at abscission-zone determinants were fundamental for the shift to reduced shattering during rice domestication.

Beyond shattering, additional domestication-related *loci* have been implicated in remodellingrice architecture. The semi-dominant gene *PROSTRATE GROWTH1* (*PROG1*), which encodes a Cys(2)-His(2) zinc-finger protein, is described to regulate upright growth, tiller number, and panicle architecture, all of them advantageous traits in cultivated rice for denser planting and higher yield potential (Tan et al., 2008). Recent work places *PROG1* upstream of shoot gravitropism and panicle development, revealing its pleiotropic control of plant habit and inflorescence architecture. *PROG1* regulates tiller angle by acting as a transcriptional repressor of *LAZY1* (*LA1*), gene in charge of controlling plant gravitropism and tiller angle (Li et al., 2019). Beyond tiller angle, this gene also influences panicle architecture through Os-*GIGANTEA* (Os-*GI*) associated pathways, which affectsthe timing of flowering and the spatial patterning of panicle branches by interacting with circadian and photoperiod-responsive signalling modules (Wang et al., 2023; Zhang et al., 2023). Population-genetic analyses indicate selection on PROG1 from standing variation within the *O. rufipogon* ancestor, with the domesticated allele(s) subsequently fixed across *indica* and *japonica* backgrounds (Lu et al., 2022).

Colour traits have also been major targets of selection during rice domestication. The *Red pericarp* (*Rc*) locus encodes a basic helix-loop-helix (bHLH) factor required for red pericarp pigmentation. It activates flavonoid biosynthetic genes, including *Rd*, which encodes Dihydroflavonol 4-reductase (DFR), a key enzyme controlling the accumulation of proanthocyanidins in the pericarp (Furukawa et al., 2007). The classical domestication allele carries a 14-bp deletion in exon 6, producing a non-functional *rc* protein that translates into a white pericarp in most cultivated rice varieties. This allele was first characterised by Sweeney et al. (2006) (Sweeney et al., 2006) and has since been confirmed in genome-wide association (GWAS) and population-genomic studies across diverse rice germplasm (Yang et al., 2022). *LONG AND BARBED AWN1* (*LABA1*), which encodes a cytokinin-activating enzyme, specifies long, barbed awns in wild rice; a frameshift in cultivated rice lowers cytokinin in awn primordia, disrupting barbing and shortening awns. Population scans reveal a region (~800 kb) surrounding the gene with a nucleotide diversity significantly reduced in cultivated rice when compared to its wild ancestor, a possible indicator of artificial selection (Hua et al., 2015).

The domestication of *Zea mays* from its wild ancestor teosinte (*Zea mays* spp. *parviglumis*) represents another classic example of developmental reprogramming. Major morphological differences include a shift from a highly branched structure with multiple small ears to a single dominant central stalk with one or a few large ears, along with the transition from a fruit case entirely enclosed by the outer glume of the spikelet to softer, smaller glumes (Stitzer and Ross-Ibarra, 2018). A key driver of this transformation is *teosinte branched1* (*tb1*), which encodes a TEOSINTE BRANCHED1/CYCLOIDEA/PROLIFERATING CELL FACTOR (TCP) family transcription factor, responsible for regulating aerial branching through direct activation of the *TASSELS REPLACE UPPER EARS1* (*TRU1*) gene that encodes an ankyrin repeat domain protein containing a BTB/POZ motif necessary for protein–protein interactions (Dong et al., 2017). A regulatory change, caused by the random insertion of a *Hopscotch* retrotransposon approximately 60 kb upstream of the gene, translated into an enhancer that has been linked to *tb1* upregulation and consequently suppression of branch outgrowth (Dong et al., 2019).

Complementing TB1 activity is a putative downstream effector, *grassy tillers1* (*gt1*), which encodes a class I homeodomain leucine zipper (HD-ZIP I) transcription factor that suppresses the elongation of lateral ear branches and maintains bud dormancy (Whipple et al., 2011). Notably, evidence from monocots shows that both genes are transcriptionally responsible to phytochrome-dependent shade cues, classically, shifts in the far-red:red (FR:R) ratio, and to endogenous hormonal cues, among other environmental inputs (Kebrom et al., 2006; Whipple et al., 2011). Rather than acting independently, these TFs act as a regulatory hub that modulates axillary bud dormancy in response to diverse signals. During maize domestication, selection acted on regulatory variants at both *loci*, contributing to reduced branching relative to teosinte (Wills et al., 2013).

Transcriptional profiling and genome-wide occupancy in maize tiller buds have revealed that *tb1* targets numerous cis-regulatory targets, including *gt1*, forming a *tb1–gt1* module that coordinates abscisic acid (ABA), jasmonic acid (JA) and sugar-signalling networks. This regulatory framework is consistent with direct measurements of hormone and metabolite levels. In addition, *tb1* binds its own promoter and the regulatory regions of key domestication *loci*, such as the major QTL controlling prolificacy (*prol1.1*) and *teosinte glume architecture1* (*tga1*). These interactions provide a mechanistic explanation for how variation at the *tb1* regulatory hub, possibly by a *Hopscotch* enhancer, could lead to major, coordinated changes in plant architecture that were favoured during selection (Dong et al., 2019). Collectively, these data place *tb1* and *gt1* at pivotal nodes that integrate light quality, hormone status and carbon signals to switch axillary buds between dormancy and growth, thereby channelling domestication-driven regulatory changes into agronomically favourable plant architecture (Wills et al., 2013; Studer et al., 2017).

Beyond this regulatory hub, downstream effectors help translate regulatory dosage into organ-level outcomes. As noted above, *tb1* physically engages the *tga1* regulatory region; that encodes a SQUAMOSA Promoter-Binding-Protein-Like (SBP) family TF and plays a major role on the soft, exposed kernels characteristic of domesticated maize varieties. A single amino acid substitution (SAAS) in *tga1* alters glume hardening, resulting in reduced lignification and a looser fruit case. In a similar manner, *shattering1* (*sh1*), which encodes a YABBY family TF, influences seed dispersal by regulating abscission layer development. Loss-of-function alleles in *sh1* reduce seed shattering, a trait favoured for harvest efficiency (Studer et al., 2011; Lin et al., 2012).

Extending beyond rice and maize, barley provides a complementary cereal example. Row-type transitions involve regulators such as *Six-rowed Spike 1* (*Vrs1*) (an HD-ZIP I homeobox gene) that shift lateral spikelet fertility, thereby redistributing inflorescence-meristem output and altering grains per spike (Zwirek et al., 2019). As a eudicot counterpoint, tomato shows that the same meristem logic scales to fruit traits; cis-regulatory variation at the *LOCULE NUMBER* (*LC*)/*WUSCHEL* (*WUS*) locus and structural variants at *FASCIATED* (*fas*)/*CLAVATA3* (*CLV3*) resize floral meristems, shifting locule number and fruit size within a familiar *WUS*-promoting/CLV-restricting framework that intersects with cytokinin/auxin modules (Muños et al., 2011). These cases underscore that small, dosage-tuned changes at meristem nodes scale to yield traits across lineages.

Another recurring theme is the extensive use of standing genetic variation during domestication. Alleles influencing meristem-related traits often segregate at appreciable frequencies in wild populations, and local haplotype structure around domesticated *loci* frequently supports soft-sweep dynamics (Liang et al., 2021). These observations indicate that domestication and improvement commonly proceed through quantitative modulation of gene regulation rather than through *de novo* loss-of-function mutations (Swinnen et al., 2016). At the molecular level, this is achieved by retiming and relocalising gene expression via promoter polymorphisms, distal enhancer insertions, or structural variants that rewire enhancer–promoter communication without altering coding sequence (Bai et al., 2024; Ciren et al., 2024). Such cis-regulatory changes are likely to shift developmental thresholds in distinct SAM domains (central versus peripheral, axillary versus apical), thereby altering cell-fate allocation and meristem outputs with reduced pleiotropy relative to coding knockouts, consistent with general principles of regulatory evolution (Ciren et al., 2024; Li and Schmitz, 2025). In practical terms, these insights suggest that modern breeding could benefit from prioritising the discovery and deployment of favourable standing variants, for example through graded CREs editing, to fine-tune determinacy and architecture (Dong, 2024).

However, selection on these regulatory control points is unlikely to be neutral with respect to trade-offs: increased determinacy may enhance harvest index at the expense of architectural plasticity, erect ideotypes suited to dense planting can reduce tillering or modify internode elongation, and loss of dispersal traits, while improving harvest efficiency, may compromise resilience in low-input environments (Dwivedi et al., 2021). Recognising and explicitly managing these constraints will therefore be essential for engineering strategies that aim to balance yield with robustness through domain-specific, dose-controlled regulatory interventions.

Taken together, domestication phenotypes are most parsimoniously explained by sustained selection on transcription factors (TFs) and their cis-regulatory elements (CREs), rather than on structural proteins. Comparative genomics and functional genetics across crops show that small changes in enhancers and promoters at dosage-sensitive regulators shift when, where and how much key genes are expressed, producing quantitative effects on meristem size, determinacy and organ allocation (Ciren et al., 2024; Li and Schmitz, 2025). Although many selected nodes sit upstream of circuits, in some lineages the circuits themselves have been tuned by regulatory alleles at meristem-size controllers. In aggregate, domestication has mostly adjusted regulatory control points, TFs and their CREs that interface with hormone and meristem networks, to deliver favourable architectures, rather than altering downstream structural effectors.

In sum, domestication has acted principally through regulatory rewiring at dosage-sensitive nodes embedded in conserved meristem networks, reshaping inflorescence architecture, abscission and branching in predictable ways. Because many targets are conserved and mechanistically understood, strategic manipulation of orthologues, via cis-regulatory tuning, chromatin-state modulation and small-RNA circuit design, offers a rational path to adjust meristem determinacy, organ initiation and tissue differentiation with controlled pleiotropy.

## 4 Current approaches for meristem manipulation to enhance crop yield

The phenotypic traits in major crop species, consequence of domestication, exemplify how subtle regulatory mutations can profoundly reshape meristem behaviour and plant architecture. This natural example highlights the potential of targeting meristem-regulating genes to alter growth patterns. In contrast to relying on spontaneous mutations and selection, current biotechnological approaches offer precise tools to manipulate these same developmental pathways, opening new possibilities for rational crop design and trait enhancement.

### 4.1 Creation of new allelic variants through random and targeted mutagenesis

**Figure 2:**
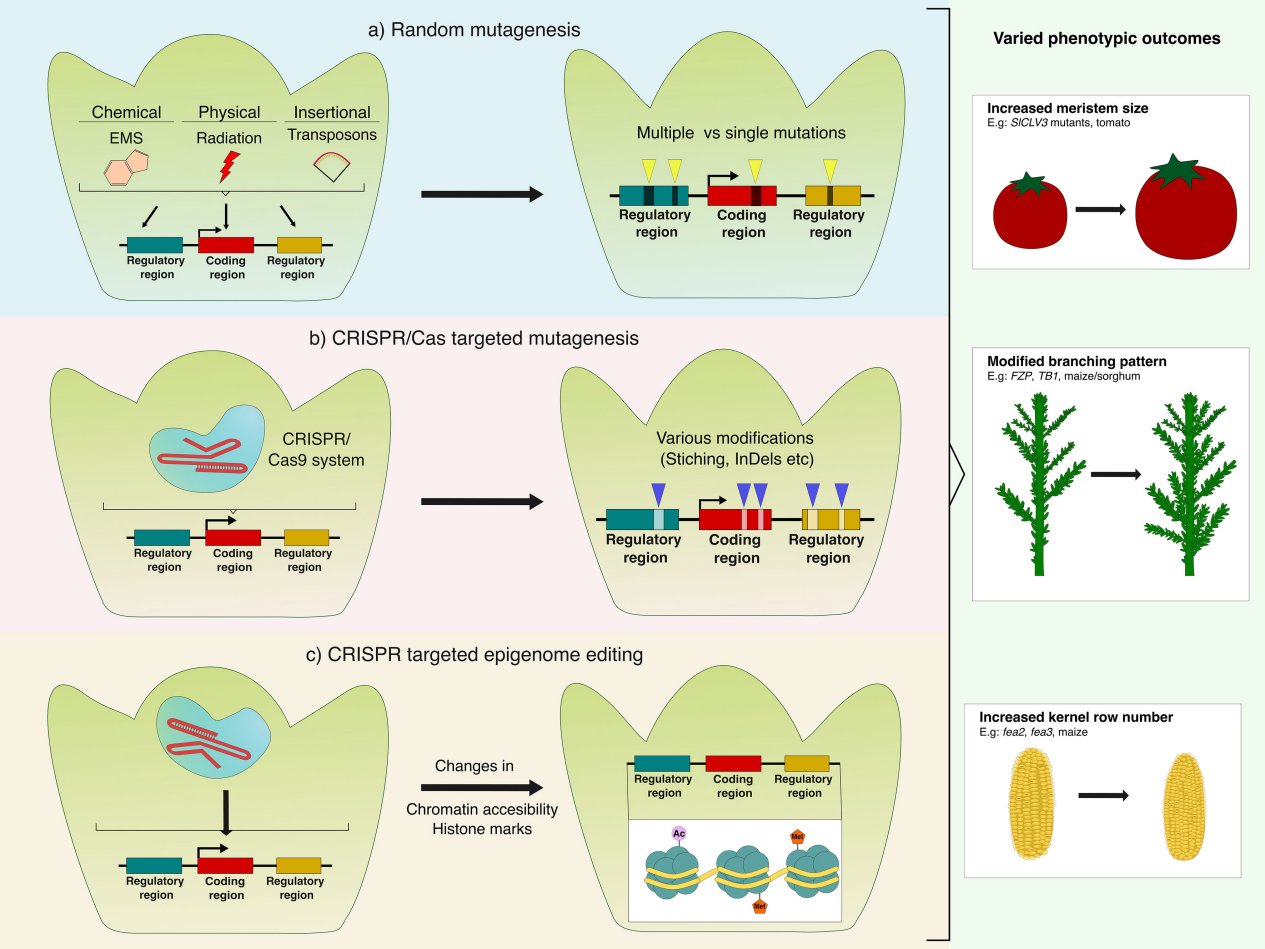
Conceptual overview of different major mutagenesis and editing approaches used to manipulate meristem regulators. **(a)** Random mutagenesis using chemical (e.g. EMS), physical (irradiation), or insertional (transposon) agents generates multiple or single mutations across coding and regulatory regions, shown by yellow arrowheads, that produce varied allelic outcomes. **(b)** CRISPR/Cas-targeted mutagenesis introduces locus-specific modifications, indicated by blue arrowheads and including but not restricted to insertions, deletions, or substitutions that enable precise tuning of developmental regulators. **(c)** CRISPR-based targeted epigenome editing modulates chromatin accessibility and histone marks without altering DNA sequence, offering reversible control of gene expression. Examples include the targeted addition of acetyl or methyl groups. These approaches can be applied to both coding sequences and cis-regulatory regions located upstream or downstream of the target gene. They generate diverse phenotypic outcomes in crops, including altered meristem morphology such as increased kernel row number (KRN) in maize or enlarged meristem size in tomato, as well as broader architectural modifications like enhanced branching in rice.

Classical mutagenesis has been a powerful entry point into the genetic control of meristem size. Among the available mutagenic approaches available, like irradiation or insertion-based methods, chemical mutagenesis, particularly with ethyl methanesulfonate (EMS), has remained one of the staple methods. EMS is an alkylating agent that predominantly induces G/C→A/T transitions, generating a high density of quasi-random, irreversible single-nucleotide variants across the genome and thereby producing a broad spectrum of allelic outcomes (**Figure 2a**) (Gillmor and Lukowitz, 2020). In contrast to the large deletions and rearrangements that arise from of irradiation (Ma et al., 2021) or the binary effects of targeted knockouts, EMS-induced hypomorphic alleles can reduce protein activity or slightly alter regulatory elements, an ideal outcome for manipulating developmental regulators that require dosage sensitivity. This mechanism has been extensively described in *A. thaliana*, where EMS-based forward genetic screens helped with isolation of the *CLAVATA* gene series (*clv1*, *clv2*, *clv3*), whose mutants exhibit progressively enlarged meristems and disrupted floral patterning (Clark et al., 1993; Clark et al., 1995; Kayes and Clark, 1998). Complementary EMS-derived *wus* mutants showed the key role of this TF in meristem maintenance, helping describe the currently well-known CLV/WUS interaction loop (Laux et al., 1996).

Building directly on this foundation, induced mutagenesis is now a practical bioengineering tool used in crops. A clear example is observed in maize, where mutagenesis of meristem regulators has yielded landmark discoveries on the genetic basis of meristem homeostasis. Major targets include *FASCIATED EAR 2* (*FEA2*) and *3* (*FEA3*) *loci*, which encode receptor-like proteins responsible for restricting stem cell proliferation at the IM when perceiving specific CLAVATA3/EMBRYO SURROUNDING REGION (CLE) peptides (Schlegel et al., 2021). EMS-derived weak alleles of these genes produce moderately enlarged meristems that translate into increased kernel row number (KRN), a quantitative trait directly linked to yield, without disadvantageous traits characteristic from null alleles such as reduced ear length (Bommert et al., 2013; Je et al., 2016). This moderate phenotype establishes the idea of a partial reduction in sensitivity to CLE peptides, such as ZmCLE7 and ZmFCP1 transduced by *FEA2* or ZmFCP1 by *FEA3*. Beyond maize, induced mutagenesis coupled to mapping-by-sequencing and TILLING (Targeting Induced Local Lesions IN Genomes) has become routine in cereals and solanaceous crops, enabling targeted recovery of allelic series in meristem regulators (Mo et al., 2018; Jiang et al., 2022). For example, *FON1* and *FON2* in rice, *CLV/WUS* homologues in tomato, and expanding EMS resources in wheat collectively demonstrate that modest modulation of *CLV–WUS* signalling can be exploited across diverse crop lineages.

However, mutagenic approaches face limitations, including random location positioning, introduction of undesirable mutations or pleiotropy from constitutive overexpression. Consequently, these techniques are now typically used as a complement to other targeted precision breeding tools like Clustered Regularly Interspaced Short Palindromic Repeats (CRISPR)/CRISPR Associated (Cas) Systems (**Figure 2b**). The value of this system mainly resides in its capacity to create locus-specific edits that enable precise modulation of meristem regulatory networks, for instance by fine-tuning the expression or dosage of *CLV–WUS* pathway components. This technology fundamentally works by generating double-stranded breaks (DSBs) at specific sites that are later repaired by the host machinery, generally via non-homologous end joining repair (NHEJ) but sometimes by microhomology-mediated end joining (MMEJ/alt-EJ) or homology-directed repair (HDR), to produce nucleotide insertions/deletions (InDels) (Belhaj et al., 2015; Chen et al., 2022). CRISPR-based editing has been extensively used in crops to enhance desirable traits. Knockdown of *Grain Size 3* (*GS3*) in rice resulted in the enhancement of grain length by 31.39% and consequently in 1000-grain weight by 27.15% (Usman et al., 2021). Multiplex gene editing techniques targeting *GS3* along with *GW2*, and *Gn1a*, also known to negatively regulate yield phenotypes like size, width and weight, resulted in significant yield increase in the mutated varieties (Zhou et al., 2019). Similar studies in hexaploid wheat using CRISPR/Cas9 with tandem tRNA–gRNA cassettes, also showed a significant increase in seed size and grain weight by delivering mutations to the *GW2*, *Lpx-1*, and *MLO* genes (Wang et al., 2018).

Regarding targeted modification of plant architecture, current advances in gene editing technology allow to induce changes with reversible effects in genes directly correlated with these phenotypic traits. Genes controlling panicle regulation, which plays a crucial role in determining the number of grains per plant and therefore directly influences grain yield, have been identified in the past decades (Li et al., 2021). For instance, using a CRISPR/Cas9 system to mutate *DENSE AND ERECT PANICLE1* (*DEP1*) allows the creation of alleles that translated into yields surpassing the phenotype conferred by other naturally present high-yield alleles in rice (Huang et al., 2009). In another study, by using similar CRISPR/Cas9 technology to precisely disrupt *CAROTENOID CLEAVAGE DIOXYGENASE 7* (*CCD7*), key for inhibiting shoot branching because of its role in strigolactone (SL) biosynthesis, resulted in a significant enhancement of tillering compensated by a decrease in plant height (Butt et al., 2018). A comparable outcome was observed when targeting the homologue gene in grapevine (*VvCCD8*) (Ren et al., 2020). Plant height is also a trait of interest in the context of crop yield, due to its correlation with biomass and node spacing. The *sd1* mutant in rice, that results in ‘Green Revolution’’ semi-dwarf phenotype, was recreated using CRISPR/Cas9 to generate loss-of-function alleles of the *OsGA20ox2* gene, implicated in gibberellin (GA) plant growth hormones biosynthesis. The outcome reported a reduction in plant stature of ~22% and a yield increase of ~6% without compromising other agronomic traits (Han et al., 2019).

CRISPR tuning of domestication *loci* and developmental regulators shows complementary routes to sturdier, higher-yielding rice. For instance, CRISPR/Cas9 mutagenesis of the seed-shattering QTL *qSH1* generated transgene-free *indica* lines with significantly reduced shattering and no obvious penalties in other measured agronomic traits; crossing these parents yielded hybrids with intermediate, harvest-friendly shattering (Sheng et al., 2020). Finally, another significant outcome arose from the multiplex editing of two important regulators of grain size and plant architecture: *MIR396e* and *MIR396f*. Mutations of these genes (*mir396ef*) exhibited enhanced grain yield and flag leaf area, which was attributable to an elevated concentration of mevalonic acid, a gibberellin precursor (Miao et al., 2020).

### 4.2 Precision editing of regulatory sequences

Despite the described evidence supporting positive phenotypic traits by using targeted genome editing (GE) approach in protein-coding regions, recent advances have shifted the attention to the non-coding regulatory genome (**Figure 2b**). Core meristem regulators and their representative orthologues are summarised in **Table 1**. When targeting cis-regulatory elements (CREs), including promoters, enhancers, untranslated regions (UTRs), and insulators; it becomes possible to modulate the quantitative and spatio-temporal dynamics of gene expression. Thus, target editing these regions provides an interesting route to fine-tune transcriptional outputs without compromising essential protein coding functions, a particularly advantageous strategy for developmental regulators whose dosage must be precisely balanced (Svitashev et al., 2015; Saeed et al., 2022). Conventional CREs modification entail strategies like building recombinant (chimeric) promoters, via promoter swaps, tissue-specific or inducible control, and enhancer/motif stacking with defined spacing. Additionally, T-DNA activation tagging provides gain-of-function up-regulation to probe or exploit regulatory dosage, as shown in rice where activation of *CYP734A4* increased grain number per the main panicle and seed setting rate (Qian et al., 2017).

**Table 1:** Core meristem regulators and representative orthologues.

Species	Functional module	Gene name	Reference ID	Representative orthologues	Activity in meristems
*Arabidopsis thaliana*	Stem-cell organising centre	*WUSCHEL (WUS)*	AT2G17950	Rice: WOX-related (varies by study)	Promotes organising-centre identity and sustains stem-cell fate
				Maize: WOX-related	
	Stem-cell restriction (CLE ligand)	*CLAVATA3 (CLV3)*	AT2G27250	Rice: *FON2/FON4* (Os11g0595400)	Secreted CLE signal restricting meristem size via receptor pathways
				Maize: CLE ligands (e.g. *ZmCLE* family)	
	CLE receptor kinase	*CLAVATA1 (CLV1)*	AT1G75820	Rice: *FON1* (Os06g0717200; LOC_Os06g50340)	Perceives CLE signals; limits stem-cell domain and meristem proliferation
				Maize: *TD1* (Zm00001d021145; also described as Zm00001eb318390)	
	CLE co-receptor component	*CLAVATA2 (CLV2)*	AT1G65380	Rice: CLV2-like genes (multiple)	Receptor complex component; constrains overproliferation of meristematic cells
				Maize: *FASCIATED EAR2* (*FEA2*); receptor-like protein	
	KNOX-mediated meristem maintenance	*SHOOT MERISTEMLESS (STM)*	AT1G62360	Rice: *OSH1* (Os03g0727000; LOC_Os03g51690)	Maintains indeterminacy; promotes CK-associated meristem maintenance programmes
				Maize: *KN1* (KNOTTED1; conserved class I KNOX)	
*Zea mays*	Meristem size limitation	*FASCIATED EAR4 (FEA4)*	Zm00001eb280500 / Zm00001d037317	Rice: bZIP orthologues (multiple)	Restricts proliferation; promotes peripheral differentiation (context-dependent)
	Inflorescence meristem determinacy	*RAMOSA3 (RA3)*	Zm00001eb327910 / Zm00001d022193	Rice: TPP family (multiple)	Trehalose-6-phosphate phosphatase; shapes determinacy/branching outputs
	Axillary branching repression	*TEOSINTE BRANCHED1 (TB1)*	Zm00001eb054440 / Zm00001d033673	Rice: *OsTB1/FC1* (LOC_Os03g49880; RAP-DB Os03g0706500)	Represses axillary outgrowth; integrates hormonal and developmental cues
*Oryza sativa*	Spikelet meristem identity/determinacy	*FRIZZY PANICLE (FZP)*	Os07g0669500	Maize: *BD1* (branched silkless1; model IDs reported across assemblies)	Enforces spikelet fate; suppresses reiterative branching in developing inflorescences

Gene IDs were obtained from public databases, TAIR for *Arabidopsis thaliana*, MaizeGDB for *Zea mays* and RAP-DB (Rice Annotation Project Database) for *Oryza sativa*.

The positive outcomes of CREs editing have been demonstrated in species like tomato, where CRISPR was used to target the promoter of *CLV3*, gene known to play a central role in the *CLV-WUS* feedback loop. This approached generated diverse cis-regulatory alleles translated into a wide range of phenotypes, an ultimate result that helps address the issue of breeding diversity (Rodríguez-Leal et al., 2017). Another interesting effect was described in tomato when editing the AGAMOUS-bound CArG motif at the *SlWUS* locus. Corresponding to the locule number (*lc*) QTL. Disruption of this CRE likely weakens *AGAMOUS* leading to increased *SlWUS* expression and a consequent increase in locule number (Li et al., 2017). Building on this paradigm, promoter editing has also been used to recalibrate meristem activity and inflorescence complexity in other crop species. In maize, the creation of weak promoter alleles of this *CLV3* orthologue: *ZmCLE7*, together with a null allele of the partially redundant compensating gene *ZmCLE1E5*, modestly enlarges inflorescence meristems and increases yield traits of interest like kernel-row number or kernel number per row, demonstrating that quantitative tuning of CLE dosage can boost productivity without gross fasciation (Liu et al., 2021). The resulting line increased both tiller number and panicle size and delivered higher field yield; mechanistic analyses traced the deleted fragment to a binding site for *Anther ear1* (*An-1*), a basic helix-loop-helix transcription factor that represses *IPA1* expression in panicles and roots (Song et al., 2022).

In barley, editing the GCN4/Skn1/RY motif in the promoter region of *HvPAPhy_a* (*Purple Acid Phosphatase phytase a*), the gene predominantly responsible for mature grain phytase activity (MGPA), reduced MGPA significantly. Edited CRISPR lines showed very low levels of MGPA, even lower when the editing event was downstream the motif. This confirms PAPhy_a as the main regulator and makes the gene a promising target for crop yield improvement by enhancing emergence and phosphorus-use efficiency on low-P soils, which can translate into more uniform stands, greater ear density, and more stable grain yield (Holme et al., 2017). Beyond these examples, additional promoter editing studies in rice have further demonstrated the potential of CRE manipulation to enhance agronomic performance. For instance, editing in rice *nicotianamine synthase 2* (*OsNAS2*) promoter, particularly the CREs deletion of ARR1AT at position −933 reported enhanced Zn in the grain and per plant. Evidence showed that this feature ultimately translated in an improved spikelet number per main panicle and therefore increased grain per plant (Ludwig et al., 2024). Another recent rice study also demonstrated that deletion of a 54 bp promoter CRE at IPA1 and a CRE3 region quantitative trait locus for panicle length on chromosome 6 (qPL6) increased yield by 23–41% across two seasons (Zhang et al., 2025). Together, these examples illustrate that regulatory-sequence engineering represents a broadly deployable strategy to modulate dosage of developmental and yield-related genes. Precise edits at the 3′ end of LONELY GUY-like 5 (*OsLOGL5*), a cytokinin-activation gene, increased grain yield across contrasting field conditions. In contrast, constitutive *OsLOGL5* overexpression produced the opposite outcome, resulting in reduced primary root growth, fewer tillers, and lower yield, underscoring the value of allele engineering over blanket up-regulation (Wang et al., 2020).

Despite rapid advances in targeted editing, the discovery of CREs controlling key genes remains a pressing priority. Even though multiple genes associated with agronomically important traits are already described, tools like genome-wide association studies (GWAS) are powerful methods to further elucidate additional regulatory targets for crop improvement. When CREs associated with important traits are found, it becomes a much simpler and time-efficient task to create variations in a target trait. For instance, a pan-genomic analysis of 66 rice accessions helped catalogue 23 million sequence variants in the genome and identified that each gene contains multiple coding variants in several haplotypes and non-coding regions, including promoters (Zhao et al., 2018). This suggests that variations in promoter regions play a potential role in regulating gene expression. Other examples of promoters identified through GWAS in major crops include those associated with *Oryza sativa SUPERNUMERARY BRACT* (*OsSNB*) and *Grain Size on Chromosome 5* (*GSE5*) (Duan et al., 2017; Ma et al., 2019), linked to grain size and ultimate yield production; or *Zea mays Trehalase 1* (*ZmTre1*) in maize (Wen et al., 2018), correlated with grain quality.

In addition, given that CRE function is tightly coupled to chromatin accessibility, the need for tools to identify open chromatin regions (OCR) becomes evident. Assays such as transposase-accessible chromatin using sequencing (ATAC-seq) and micrococcal nuclease digestion with deep sequencing (MNase-seq) enable genome-wide identification of accessible regulatory DNA (Parvathaneni et al., 2020; Sun et al., 2020). Another level of complexity can be included in these studies if implementing the current available technology to achieve single-cell resolution. In combination with ATAC-seq, a new study has identified cell-type-specific CREs in maize, that were enriched for enhancer activity and within unmethylated long terminal repeat retrotransposons. Notably, these sequences were also proven to be hotspots for phenotype-associated genetic variants, targeted by selection during breeding (Marand et al., 2021).

### 4.3 Epigenetic modulation of chromatin accessibility

While CRISPR editing of cis-regulatory elements provides durable modifications to transcriptional activity, an emerging frontier in meristem engineering lies in epigenetic fine-tuning. This strategy relies on targeted modulation of chromatin states to control the accessibility and responsiveness of regulatory *loci* (**Figure 2c**). Epigenetic regulation, including histone modifications, chromatin remodelling and DNA methylation, adds a dynamic and reversible layer to gene control, allowing transient yet heritable adjustments of transcriptional potential without altering DNA sequence (Zhang et al., 2022). Recent advances in locus-specific chromatin engineering now allow direct manipulation of these marks using epigenetic editors. By tethering catalytically inactive Cas9 (dCas9) to histone modifiers, such as acetyltransferases, methyltransferases, or demethylases, chromatin states can be rewritten at individual meristem genes *in planta*.

In a recent proof-of-concept study Fal et al. (2025) used a CRISPR-dCas9 SUperNova (SunTag) system to remove repressive trimethyl mark deposited by JUMONJI 13 (JMJ13) at Lysine 27 of Histone 3 (H3K27me3) at the *CUP-SHAPED COTYLEDON3* (*CUC3*) locus in *Arabidopsis*. This targeted delivery led to enhanced expression and altered organ boundary formation. These studies underscore that targeted histone demethylation can reprogramme developmental gene expression in plants (Fal et al., 2025). Similarly, Gallego-Bartolomé et al. (2018) showed that by using a system that fuses the catalytic domain of the human demethylase TEN-ELEVEN TRANSLOCATION1 (TET1cd), referred to as SunTag–TET1cd. When targeting the promoter of the *FLOWERING WAGENINGEN* (*FWA*) gene, TET1 induces stable local DNA demethylation, generating novel epialleles that persisted across generations without sequence modification (Gallego-Bartolomé et al., 2018). Together, these studies demonstrate that targeted chromatin editing can stably reprogramme developmental gene expression in plants, establishing the feasibility of “programmable epialleles” as a new class of regulatory variants for crop improvement.

In the context of meristem function, key regulators such as *WUS* and *KNOX* genes like *STM*, necessary for stem cell niche maintenance, are dependent on epigenetic marks that collectively balance activation and repression within the SAM (Guyomarc’h et al., 2005). This example was demonstrated when directly targeting *FASCIATA1* (*FAS1*) and *FASCIATA2* (*FAS2*), two subunits of the CHROMATIN ASSEMBLY FACTOR 1 (CAF-1) complex in *Arabidopsis thaliana*. This protein aggregation is described to ensure stable propagation of epigenetic states, and when mutated, led to aberrant SAMs with abnormal *WUS* expression (Kaya et al., 2001).

However, this pathway has not yet been directly targeted in *A. thaliana* to modulate meristem phenotypes. A promising direction would involve the use of targeted epigenetic tools to manipulate histone marks and chromatin accessibility in key regulatory regions. In mammalian systems, for instance, dCas9–p300 is a fusion to the catalytic histone acetyltransferase domain of E1A-binding protein p300 (EP300). This construct can be targeted to specific *loci* to locally increase histone 3 acetylation at lysine 27 (H3K27ac) thereby promoting transcriptional activation. By contrast, dCas9–DNMT3A is a fusion to DNA methyltransferase 3 alpha and can deposit DNA methylation at defined genomic sites to achieve stable transcriptional repression. Adapting these tools to meristem regulators could, in principle, enhance stem-cell activity by increasing activating histone acetylation at *WUS* regulatory regions while reinforcing feedback repression through targeted DNA methylation at *CLV3*. Similar approaches in plants include dCas9–KRAB (Krüppel-associated box), used in *Nicotiana* to repress target gene expression by recruiting endogenous silencing machinery (Hilton et al., 2015; Liu et al., 2016; Alok et al., 2025).

Although targeted epigenome editing of meristem-specific genes has not yet been translated to major crops, epigenetics-mediated crop breeding (epibreeding) is emerging as a strategy to engineer agronomically desirable traits by coupling CRISPR/Cas-based systems with Synthetic Epigenetics (SynEpi). Programmable systems such as CRISPRoff and CRISPRon use guide RNAs to recruit catalytically inactive Cas (dCas) effectors that, install or erase DNA methylation at desired *loci*, enabling durable silencing or reactivation without changing the DNA sequence (Nuñez et al., 2021). Likewise, SunTag–DRM modules multimerise dCas-bound methyltransferase domains to drive *de novo* methylation at target promoters or enhancers.

Proof-of-concept gains have also been reported via epitranscriptome editing. In rice and potato, transgenic expression of the human FTO protein, originally identified as a fat mass- and obesity-associated protein, functions as an N^6^-methyladenosine (m^6^A) RNA demethylase. Its expression has been reported to reduce global m^6^A levels, increase poly(A) RNA abundance and chromatin accessibility, and enhance stress tolerance and yield (Yu et al., 2021). On the DNA side, editing in rice cells with a CRISPR–Cas12j2 5mC down-regulated *Oryza sativa GRANULE-BOUND STARCH SYNTHASE I* (*OsGBSS1*) has been performed, illustrating precise control of grain-filling pathways relevant to yield and quality (Liu et al., 2022). In maize, the epibreeding framework have prioritised yield-component regulators already tied to field traits, including *REPRESSOR OF SILENCING 1a* and *1b (ZmROS1a/ROS1b*), linked to kernel weight, and Histone Deacetylase 108 gene (*ZmHDA108*), related to plant height, as candidate loci for targeted methylation or histone-mark editing (Forestan et al., 2018; Xu et al., 2022).

Despite its promise, epibreeding faces practical constraints. First, specificity remains a challenge dCas–based writer or eraser fusions can deposit marks beyond the intended target window trough spillover, spreading, or recruitment of endogenous chromatin complexes, and unintended reactivation of previously silenced *loci* is a real risk (Papikian et al., 2019). This is particularly relevant for transposable elements (TEs), whose repression depends on coordinated DNA methylation and histone modifications, affecting this balance could destabilise silencing and trigger ectopic transcription (Cui et al., 2013; Qi et al., 2023). Second, stability: histone and DNA-methylation states can reset across developmental transitions, meiosis, or environmental stress, so a mark that’s established in callus or somatic tissues may not be present in the germline (Zhong et al., 2013; Harris et al., 2023). Third, outcomes are often context-dependent: DNA methylation, histone marks, and 3D genome architecture are interlocked, so editing one layer can yield non-linear outcomes at the pathway level (He et al., 2024; Zhao et al., 2025). These constraints highlight the importance of integrating epigenome editing with approaches that directly interrogate and, potentially, reconfigure higher-order regulatory organisation.

In this regard, chromatin-conformation methods have made regulatory architecture experimentally tractable and provide a logical endpoint for precision control of meristem gene regulation. Hi-C provides genome-wide contact maps, promoter-capture Hi-C enriches for promoter-centred interactions to reveal candidate distal enhancers, and Micro-C resolves fine-scale nucleosome-level loops and domain boundaries in plants (Sun et al., 2024; Wang et al., 2024). Taken together, these 3D maps show that meristem regulators sit within networks of promoter–distal contacts that likely titrate expression in space and time. A concrete exemplar is *WUS* in *Arabidopsis*, where a defined repressive loop has been functionally perturbed by CRISPR edits to loop elements, resulting in changes in its transcription pattern and floral-meristem determinacy (Guo et al., 2018). Looking ahead, programmable loop engineering, using dCas9 tethered to architectural or insulator modules, as shown by Chromatin Looping by dCas9 (CLOuD9) and Light-Activated Dynamic Looping (LADL) systems in mammalian cells, offers a plausible route to redirect enhancer–promoter contacts and quantitatively tune meristem outputs once adapted to plants (Kim et al., 2019).

## 5 Discussion

The developmental and evolutionary insights into meristem regulation presented in this review highlight how subtle molecular adjustments at conserved regulatory nodes, such as the *CLV–WUS*, *KNOX*–cytokinin and *RAMOSA*–FZP networks, have repeatedly shaped plant architecture and consequently yield throughout domestication. Thus, the existent delicate crosstalk of the molecules within these pathways shows how by focusing on incremental, dosage-sensitive changes in transcriptional and hormonal balance, rather than major coding mutations, underpin most of the morphological innovations that define modern crops. Understanding and re-engineering these quantitative regulatory systems therefore remain central to the rational improvement of crop productivity.

Recent technological advances now enable this goal to be pursued with unprecedented precision. CRISPR-based genome and epigenome editors, together with single-cell and 3D chromatin mapping, are redefining how meristem states can be manipulated in space and time. Some next promising steps could rely on integrating *in silico* prediction with *in planta* control. The current focus on developing Artificial intelligence tools and deep-learning frameworks such as the eRice database, SMOC (SMart model for Open Chromatin), and SMEP (Smart Model for Epigenetics in Plants) are beginning to decode the regulatory grammar of chromatin in crops, identifying promoter–enhancer interactions and epigenetic motifs that fine-tune gene expression at meristematic loci. When coupled with SynEpi technologies, such as inducible or split dCas-based editors carrying plant-optimised histone or methylation modifiers, these tools provide a foundation for constructing programmable epialleles capable of reversible, tissue-specific regulation of developmental genes.

Technical priorities for this emerging field include confining editor activity through short targeting windows, inducible systems, and orthogonal multiplexing (e.g. Cas12a crRNA arrays or SunTag scaffolds), as well as establishing rigorous epigenetic quality control via locus-resolved methylation sequencing, CUT&Tag and accessibility assays. Together, these strategies will allow predictable, mark-aware interventions in meristem networks.

In the long term, the convergence of AI-guided cis-regulatory discovery and SynEpi engineering will enable epibreeding: a predictive, reversible, and dosage-aware approach to plant improvement. By integrating chromatin architecture, transcriptional logic and the ability to target genome editing to desired *loci*, it will become possible to design resilient meristem programmes that improve crops’ yield and adaptability under the dynamic conditions of future agriculture.
